# Dermatan Sulfate Affects Breast Cancer Cell Function via the Induction of Necroptosis

**DOI:** 10.3390/cells11010173

**Published:** 2022-01-05

**Authors:** Grzegorz Wisowski, Adam Pudełko, Krystyna Olczyk, Monika Paul-Samojedny, Ewa M. Koźma

**Affiliations:** 1 Department of Clinical Chemistry and Laboratory Diagnostics, Faculty of Pharmaceutical Sciences in Sosnowiec, Medical University of Silesia, Katowice, Jedności 8, 41-200 Sosnowiec, Poland; adam.pudelko@sum.edu.pl (A.P.); olczyk@sum.edu.pl (K.O.); mkozma@sum.edu.pl (E.M.K.); 2 Department of Medical Genetics, Faculty of Pharmaceutical Sciences in Sosnowiec, Medical University of Silesia, Katowice, Jedności 8, 41-200 Sosnowiec, Poland; mpaul@sum.edu.pl

**Keywords:** dermatan sulfate, breast cancer cells, necroptosis, oxidative stress

## Abstract

Dermatan sulfate (DS) is widespread in the extracellular matrix (ECM) of animal tissues. This glycosaminoglycan is characterized by a variable structure, which is reflected in the heterogeneity of its sulfation pattern. The sulfate groups are responsible for the binding properties of DS, which determine an interaction profile of this glycan. However, the detailed role of DS in biological processes such as the neoplasm is still poorly understood. The aim of the study was to assess the effects of the structural variants of DS on breast cancer cells. We found that DS isoforms from normal and fibrotic fascia as well as from intestinal mucosa were able to quickly induce oxidative stress in the cytoplasm and affect the mitochondrial function in luminal breast cancer cells. Moreover, the variants caused the necroptosis of the cells most likely via the first of these mechanisms. This death was responsible for a reduction in the viability and number of breast cancer cells. However, the dynamics and intensity of all of the DS variants-triggered effects were strongly dependent on the cell type and the structure of these molecules. The most pronounced activity was demonstrated by those variants that shared structural features with the DS from the tumor niche.

## 1. Introduction

Dermatan sulfate (DS) is a component of glycoproteins termed DS proteoglycans (PG), which are commonly spread in the extracellular matrix (ECM) of the majority of animal tissues. Because of the composition of the disaccharide units, which build its po-lymeric linear chains, DS belongs to the chondroitin/dermatan subclass of the sulfated glycosaminoglycan (GAG) family. In fact, DS is a copolymer of two types of disaccharide units from which one is composed of N-acetylgalactosamine (GalNAc) and glucoronate (GlcA) residues while the other contains GalNAc and iduronate (IdoA) residues [1]. The former unit is not only present in DS but also in the chondroitin sulfate (CS) chains, in which it is the only structural element. In turn, the latter disaccharide is characteristic only for DS and is responsible for the great flexibility of chains of these GAG chains because of the more conformational elasticity of IdoA residue compared to its C5 epimer—GlcA one [2]. Another structural feature of DS, which is also shared with the remaining sulfated GAGs such as heparin, heparan sulfate, and keratan sulfate, is the sulfation of saccharide residues in the chains of this GAG. This modification that concerns the majority of GalNAc residues, which can have a sulfate group in a position at 4 and/or 6 C, and some hexuronate ones, in which this group is localized at 2 C, is responsible for the high density of negative electric charge in the DS chains and for the ability of these chains to interact via ionic bonds with miscellaneous protein ligands, for instance, many cytokines and growth factors, the so-called structural components of the ECM or various cell surface receptors [3]. Therefore, DS can not only regulate the ECM structure and properties but also the behavior of cells. Most probably there is a tissue-specific binding profile of DS that is associated with the tissue-specific functions of this GAG, which is accomplished by the tissue-specific composition of its chains. Importantly, the DS structure can even undergo marked remodeling during many physiological and pathological processes, which is reflected in changes in both the number and chain backbone localization of the sulfate groups and IdoA residues. However, a relationship between the structure of DS and its biological properties as well as the detailed role of this compound in the biological processes are still poorly understood.

There is a significant remodeling of the DS (and CS) metabolism in the cancer microenvironment [4]. These alterations are most often manifested as an accumulation of these glycans, which is also accompanied by a progressive increase in the ratio between CS and DS compared to normal tissue. Moreover, both GAGs have an increased content of unsulfated and 6-O-sulfated disaccharides as well as a smaller chain size. In addition, the enhanced contribution of disaccharides with GlcA residue into the composition of the DS chains compared to normal tissue has also been reported. The structural remodeling of DS co-exists with the intense degradation of DSPGs that is promoted by the proteases and hyaluronidases, which are secreted from various cells that settle into the tumor niche. This degradation is an abundant source of free DS chains that reach the cancer cell surface. A similar phenomenon also occurs during metastasis. However, neither the processes that are triggered in cancer cells by this influx of DS nor the relevance of these processes for cancer progression are not fully understood. A substantial role in the stimulation of cancer growth and metastasis is attributed to chondroitin-4-sulfate chains, which are components of cancer cell surface proteoglycans, and which often contain highly reactive 4,6-O-disulfated disaccharides [5]. Nevertheless, the involvement of DS in the regulation of cancer growth and mobility has also been suggested by both in vitro and in vivo studies using a modulated expression of dermatan epimerase-1 (DSE-1), which is a key enzyme that is responsible for the biosynthesis of this GAG [2]. The down-regulation of the enzyme reduced the HGF-dependent migration and invasion of esophagus squamous cancer cells [6]. DSE-1 has also been found to promote the aggressive glioma cell phenotype by enhancing the HB-EGF/ErbB signaling [7]. In addition, an increased reactivity of the Golgi apparatus in breast cancer cells with anti-DSE-1 antibodies co-existed with a high growth potential of these cells [8]. In contrast, the down-regulation of DSE-1 in hepatocellular cancer was strongly associated with the advanced tumor stages, metastases, and poor survival due to disturbed CCL5 signaling [9]. In line with this anti-cancer effect are also observations that exogenous DS when it was applied at a high concentration to cultures of several cancer lines such as melanoma [10], osteosarcoma [11], or breast cancer [12] was able to decrease the viability and/or the proliferation of these cells. Moreover, it has recently been reported that the CS/DS that was synthesized by triple-negative breast cancer cells on a specific β-D-xylopyranoside acceptor had a strong cytotoxic effect on these cells via the induction of their apoptosis [13]. These often contradictory data as to the impact of DS on tumor cells prompted us to explore the short-term processes that might be triggered in breast cancer cells by structurally different variants of this GAG. Notably, several variants that we examined (especially those from the normal and fibrosis-affected fascia) had structural features that can in some respects mimic the DS remodeling in the tumor niche. We found that these variants as well as the DS from the intestinal mucosa can quickly induce oxidative stress in the cytoplasm and affect the mitochondrial function in luminal breast cancer cells. Moreover, the variants cause the necroptotic death of the cancer cells most probably via the first of the above-mentioned mechanisms. However, both the dynamics and intensity of all of the triggered processes were strongly dependent on the cancer cell type and the structure and concentration of an individual variant. Importantly, the most pronounced biological activity was shown by those variants that shared structural features with the DS that is synthesized in the tumor niche.

## 2. Materials and Methods

### 2.1. Tissue Material

The study protocol was approved by the local Bioethics Committee of the Medical University of Silesia in Katowice. Samples of human fibrosis-affected palmar fascia were obtained from patients who underwent surgery to treat Dupuytren’s fibromatosis. The samples of normal fascia lata were collected post-mortem. The porcine skin was purchased from a slaughterhouse. All of the tissue samples were stored at −75 °C until they were homogenized.

### 2.2. Isolation and Purification of DS from the Tissue Material

The homogenized tissue material was suspended at a ratio of 1:15 in a 0.1 M TrisHCl buffer, pH 9.0 that contained 2 mM CaCl_2_ and subjected to the action of the proteolytic enzymes from *Streptomyces griseus* (Sigma-Aldrich, Saint Louis, MO, USA), which were used at a ratio of 25 U/g of dry tissue. The proteolysis was conducted at 50 °C for 24 h under agitation. Then the samples were centrifuged (36,000× *g*, 25 min., 4 °C) and the obtained tissue pellets were hydrolyzed once again followed by centrifugation. The peptide products of the proteolysis were removed from the collected supernatants by adding 100% trichloroacetic acid to the final concentration of 8% and centrifuging (25,000× *g*, 20 min., 4 °C) after a short incubation at 4 °C. The rescued non-protein tissue components were further fractionated using specific enzymatic treatment. Recombinant heparinases I—III (Amsbio, Abingdon, UK) were applied to a 0.02 M TrisHCl buffer, pH 7.5 that contained 40 mM NaCl, 4 mM CaCl_2,_ and 0.01% bovine serum albumin for 24 h at 30 °C in order to remove any heparan sulfate. The hyaluronan was eliminated in a 0.16 M acetate buffer, pH 6.0 for 18 h at 60 °C using the hyaluronidase from *Streptococcus pyogenes* (Sigma-Aldrich), whereas the nucleic acids were degraded by treating them with the recombinant endonuclease from *Serratia marcescens* (Sigma-Aldrich) in a 0.05 M TrisHCl buffer, pH 8.5 that contained 20 mM NaCl and 2 mM MgCl_2_ for 1 h at 37 °C. The DS was separated from the obtained degradation products and from the other contaminations using ion-exchange chromatography on a DEAE-Sephacel (Merck, Darmstadt, Germany) column that was equilibrated in a 0.05 M acetate buffer, pH 6.0 that contained 0.2 M NaCl. The DS was eluted with a 2 M NaCl solution after the column was pre-washed with 0.6 or 0.4 (in the case of normal fascia material) M NaCl. Then, the DS was dialyzed, lyophilized, and quantified according to the Farndale methods [14]. The purity of the obtained DS preparations was tested using the electrophoresis on cellulose acetate in 0.034 M Al_2_(SO_4_)_3_ before and after the samples had been treated with chondroitinase ABC as was previously described [15].

### 2.3. Structural Analysis of DS

The complete depolymerization of the DS variants by chondroitinase ABC from *Proteus vulgaris* (Sigma-Aldrich) was conducted in a 0.05 M TrisHCl buffer, pH 8.0 for 24 h at 37 °C. In turn, the partial degradation of these molecules with the recombinant chondroitinase AC I from *Flavobacterium heparinum* (Amsbio) was performed in a 0.05 M Tris HCl buffer, pH 7.3 for 2 h at 30 °C. Then, the obtained unsaturated disaccharides were labeled with fluorophore aminoacridone-2 and separated using reversed phase-HPLC according to the Deakin and Lyon method [16]. Chromatography was performed on a PLRP-S 300A column (4.6 mm × 150 mm; Polymer Laboratories, Varian, Shropshire, UK) equilibrated in 0.1 M ammonium acetate and run on a Varian ProStar HPLC system. The eluted disaccharides were detected using in-line fluorescence (excitation at 425 nm, emission at 520 nm). To identify the types of eluted disaccharides, their retention times were compared to those that characterize the standard disaccharides (Iduron, Cheshire, UK). A quantitative analysis of the obtained chromatograms was performed using Galaxy software (Varian).

### 2.4. Cell Cultures

All of the breast cancer cell lines (BT-483, T-47D, and HCC-38) that were used were purchased from The American Type Culture Collection. The cells were grown in an RPMI 1640 medium (Sigma-Aldrich) that had been supplemented with 100 IU/mL penicillin, 0.1 mg/mL streptomycin, and 10% fetal bovine serum (FBS, Biowest, Nuaillé, France). Only the BT-483 cells were cultured in 20% FBS. All of the cells were grown at 37 °C in 95% humidified air with 5% CO_2_.

### 2.5. Viability, Proliferation, and Cell Count Tests

The cells were seeded into 96-well plates (Corning Incorporated, NY, USA) at a density of 3000 per well. After 24 h of incubation, the cells were transferred to a growth medium containing 0.5% FBS (BT-483 and T-47D) or to a medium with no FBS (HCC-38) and left to grow for 24 h. Then, the growth medium was exchanged for one that had been supplemented with the tested DS variants. The viability of these cells was estimated using the WST-1 test (Roche, Basel, Switzerland) according to the manufacturer’s protocol. For the proliferation test, the cells were grown in the presence of DS for 24 and 48 h. The cell proliferation was evaluated using the bromodeoxyuridine (BrdU) incorporation assay (Roche) according to the manufacturer’s protocol. To evaluate the cell count using the crystal violet test, the BT-483 and T-47D cells were fixed for 15 min in 1% glutaraldehyde after washing them twice in ice-cold PBS and stained for 30 min in a 0.1% solution of crystal violet (Sigma-Aldrich). Next, the cultures were washed three times in ice-cold PBS and permeabilized in 1% Triton X-100 for 24 h under agitation after which the absorbance of the obtained solutions was measured at λ = 600 nm.

### 2.6. Immunocytochemical Analyses of Cell Death

The BT-483 and T-47D cells were seeded into eight-well glass chamber slides (Thermo Fisher Scientific, Waltham, MA, USA) and grown for up to 24 h exposed to the selected structural variants of DS. Cell death was estimated using an Alexa Fluor 488 Annexin V/Dead Cell Apoptosis Kit (Thermo Fisher Scientific) according to the manufacturer’s protocol. In some of the experiments, the cells were treated with the necroptotic inhibitor necrosulfonamide (NSA) (Tocris, Bio-Techne, Bristol, UK). To quantify the effector caspase-positive cells, the CellEvent Caspase-3/7 test (Thermo Fisher Scientific) was used accordingly to the manufacturer’s protocol. To detect the activation of the necroptotic effector—mixed lineage kinase domain-like pseudokinase (MLKL), the cells were fixed in 3.7% formaldehyde for 10 min at 21 °C and washed three times with PBS. Next, the cells were permeabilized in 0.3% Triton X-100 for 15 min and blocked in PBS that contained 3% bovine serum albumin and 0.3% Triton X-100 (PBS-Trit buffer) for 1 h at 21 °C. The cells were then incubated with the primary antibody (the monoclonal rabbit anti-phospho-MLKL (S358) antibody (#ab187091, Abcam, Cambridge, UK)), which was used diluted at 1:300 in PBS-Trit, overnight at 4 °C. This step was followed by incubation with Alexa Fluor Plus 555-conjugated goat anti-rabbit IgG (#A32732, Thermo Fisher Scientific) (1 µg/mL in PBS-Trit) for 1 h at room temperature. Images of the stained cells were captured using a Leica DMI 6000B microscope (Leica Microsystems GmbH, Wetzlar, Germany).

### 2.7. Western Blot Analysis of the MLKL Phosphorylation Level

The BT-483 and T-47D cells were seeded into a six-well plate (Corning Incorporated) and exposed to DS from the porcine intestinal mucosa. Then, after intense rinsing, the cells were lysed in an ice-cold RIPA buffer (50 mM TrisHCl, pH 7.5, 0.15 M NaCl, 0.5% Igepal CA630 (Sigma-Aldrich), 0.5% Na deoxycholate (Sigma-Aldrich), 0.1% sodium dodecyl sulfate (SDS) (Sigma-Aldrich)) that had been supplemented with protease (Mix M, Serva GmbH, Heidelberg, Germany) and a phosphatase inhibitor cocktails (Set III, Merck). The lysates were incubated for 30 min at 4 °C under agitation and centrifuged (15,000× *g*, 20 min, 4 °C). The cellular protein concentration in the obtained supernatants was measured using a Pierce BCA Protein Assay Kit (Thermo Fisher Scientific). The lysate aliquots were resolved using SDS-PAGE 4—20% MiniProteanTGX gel (Bio-rad, Hercules, CA, USA). Next, the resolved proteins were transferred onto Immobilon P membranes (Sigma-Aldrich) and probed overnight at 4 °C with the monoclonal rabbit anti-phospho-MLKL (S358) antibody (#ab187091, Abcam). This antibody was applied diluted at 1:1000 in TBST buffer (0.05 M TrisHCl buffer, pH 7.4 containing 0.15 M NaCl and 0.1% Tween 20) that was supplemented with 5% (*w*/*v*) Blot Quick Blocker (Millipore Corp., Billerica, MA, USA). Then, after an intense washing the membranes were treated for 1 h at 21 °C with peroxidase-conjugated goat anti-rabbit immunoglobulin G antibodies (#A9169, Merck), diluted at 1:12,000 in a TBST buffer that contained 5% (*w*/*v*) Blot Quick Blocker. The immunoreactive phospho-MLKL protein was visualized using a 3,3′,5,5′-tetramethylbenzidine substrate (Merck). Before probing with the glyceraldehyde-3-phosphate dehydrogenase (GAPDH) antibodies, the blots were incubated in a stripping buffer (1.5% glycine, 0.1% SDS, and 1% Tween 20, pH 2.2) for 10 min. at 21 °C and then re-blocked. Next, the blots were probed with rabbit polyclonal anti-GAPDH antibodies (#2275, Trevigen, Gaithersburg, MD, USA) that had been diluted at 1:2500 followed by a secondary antibody as was described above.

### 2.8. Fluorescence Analyses of the Mitochondrial Transmembrane Potential and Oxidative Stress

The BT-483 and T-47D cells were seeded into eight-well glass chamber slides (Thermo Fisher Scientific). The mitochondrial transmembrane potential was measured using the MitoScreen test (Becton Dickinson, Heidelberg, Germany) according to the manufacturer’s protocol. The oxidative stress in the cytoplasm was detected using the CellROXDeepRed test (Thermo Fisher Scientific) according to the manufacturer’s protocol.

### 2.9. Statistical Analysis

The data were analyzed using Statistica 13.3 application (TIBCO Software Inc., Cracow, Poland). The normality of the distribution was verified using the Shapiro-Wilk test, whereas the variance homogeneity was analyzed using Levene’s test. The data were summarized as the mean ± standard deviation (SD) or mean ± standard error of the mean (SEM). The between-group comparisons were assessed based on a one-way ANOVA and the post-hoc Tuckey’s test with *p* ≤ 0.05 as being significant. The between-group differences for the non-parametric data were estimated using the Kruskal-Wallis test by ranks with *p* ≤ 0.05 as being significant.

## 3. Results

### 3.1. Structural Characteristics of the DS Variants That Were Used

**Figure 1:**
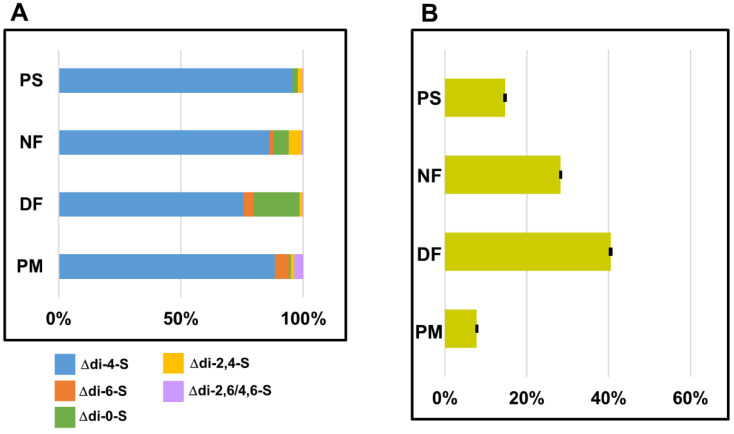
Characteristics of sulfation patterns and glucuronosyl epimerization levels in the tested variants of dermatan sulfate (DS). (**A**) Comparison of the disaccharide profiles that were obtained after the tested variants of DS were treated with chondroitinase ABC. The relative content of an individual disaccharide was calculated as a percentage contribution of the area under its peak into the total area of the chromatogram. Chromatographical analysis of sulfation pattern in DS from the porcine intestinal mucosa (PM) was conducted previously [17]. The results are expressed as the mean of two independent experiments. (**B**) Comparison of the glucuronosyl epimerization levels that characterized the tested variants of DS. This parameter was calculated as the ratio between the total areas under the peaks of the unsaturated disaccharides that were released by chondroitinase AC I and by chondroitinase ABC. The results are expressed as the mean ± SD of two independent experiments. PS—DS from porcine skin, NF—DS from the normal human fascia, DF—DS from fibrosis affected fascia.

To test the effect of DS on the function of breast cancer cell lines, we used the isoforms of this GAG, which had been isolated from several human and animal tissues. These compounds included DS from normal human fascia lata (designated as NF) and from fibrosis-affected palmar fascia due to Dupuytren’s contracture (DF) as well as DS from porcine skin (PS). Moreover, commercial DS from porcine intestinal mucosa (PM) was also used in the study. Before the cellular experiments, all of the isolated variants underwent a structural analysis that included estimating the two basic structural features of these compounds, i.e., their sulfation pattern and glucuronosyl epimerization level. The latter parameter was measured as the content of the GlcA-containing disaccharides that are assembled into blocks in the glycan chains, because such sections are a predominant form of the occurrence of this disaccharide unit in almost all of the DS chains [2]. All of the used DS variants markedly differ in respect to their composition as results from specific profiles or quantities of unsaturated disaccharides that were rescued from these molecules by chondroitinase ABC or chondroitinase AC I treatment, respectively (Figure 1 and Supplementary Figure S1). Notably, NF and especially DF was distinguished by a high content of both unsulfated disaccharide units and GlcA-containing disaccharides that form blocks (Figure 1). Interestingly, these structural features resemble the structural remodeling of DS in the cancer microenvironment to some extent. Moreover, DF has a high content of 6-O-sulfated disaccharides (Figure 1), which are also accumulated in the DS from the tumor niche. Thus, based on these structural traits of NF and especially DF, it can be assumed that both variants can mimic the biological activity of DS in the cancer microenvironment to some extent.

### 3.2. Short-Term Effect of the DS Variants on the Viability and Cell Number in Cultures of Breast Cancer Cells

**Figure 2:**
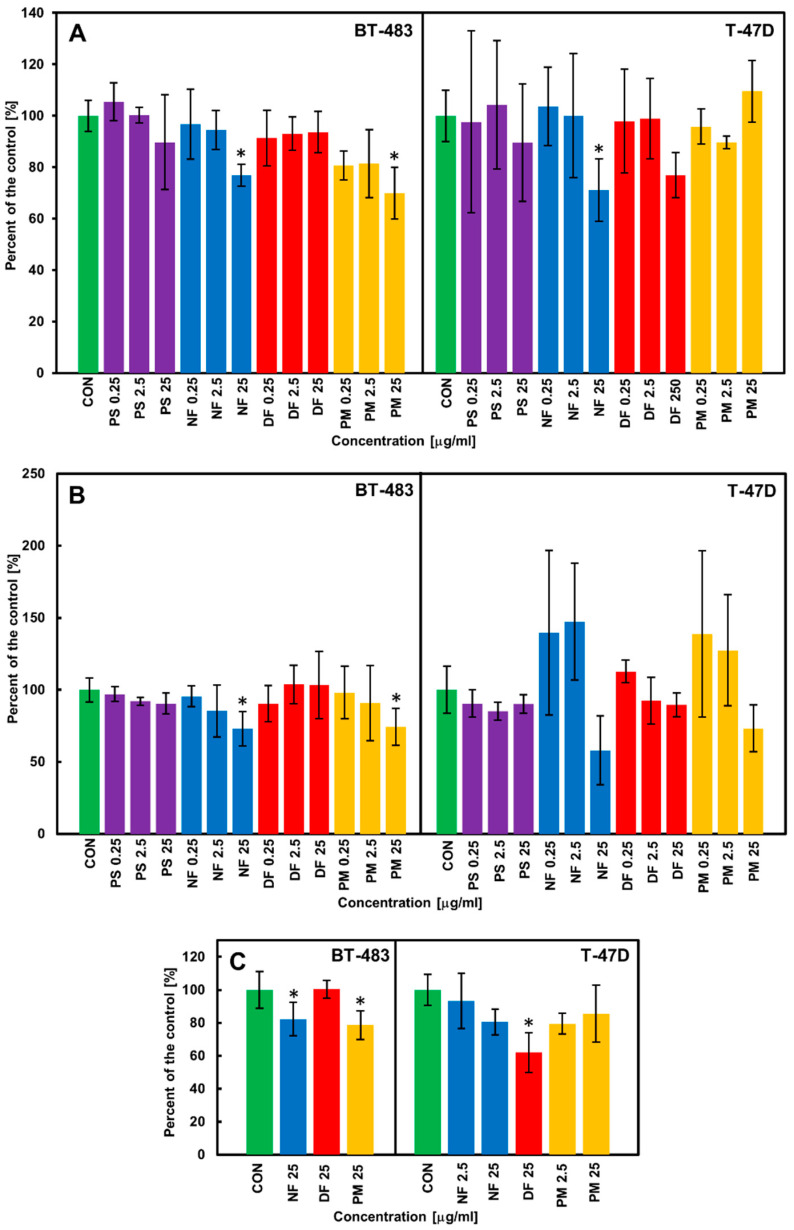
The DS variants affect the viability (**A**), DNA biosynthesis (**B**), and cell counts (**C**) of luminal breast cancer cells (BT-483 and T-47D) in a manner that is dependent on their structure and cell type. (**A**) The breast cancer cells were exposed to the structural variants of DS that had been applied at the indicated concentrations for 24 h. Then, the cell viability was evaluated using the test based on measurements of the activity of the mitochondrial dehydrogenases. (**B**) The cancer cells were grown for 48 h in the presence of the tested variants of DS that had been used at the indicated concentrations. The cell proliferation was evaluated by measuring the incorporation to DNA of the bromodeoxyuridine that had been added to the cultures after the first 24 h of incubation. (**C**) The cancer cells were grown for 48 h in the exposure to the selected DS variants that had been applied at the indicated concentrations. Then, the cell number in the cultures was estimated using the crystal violet test. The results are expressed as the percentage of effect that was visible in the control cultures and are presented as the mean ±SD of at least three independent experiments in which *n* = 3 for each DS concentration. *—difference statistically significant (*p* ≤ 0.05) versus the control. CON—control (cultures untreated), PS—DS from porcine skin, NF—DS from normal human fascia, DF—DS from fibrosis affected fascia, PM—DS from porcine intestinal mucosa.

The biological effects of the structurally distinct DS variants were examined in three lines of breast cancer cells: a luminal type primary BT-483, a luminal type metastatic T-47D, and a triple-negative HCC-38, which differed in respect to their aggressiveness. These cells were cultured via their exposure to the tested isoforms, which were applied at three concentrations that covered a wide range of values. We selected 24 h as the basal time conditions of the viability test in the luminal cancer cell lines because prolonging the incubation period to 48 h led to a significant reduction in the obtained absorbance signal in both control and treated cultures of these cells (especially T-47D). In turn, the viability of the HCC-38 cells was examined at both time periods. As can be seen in Figure 2A and Supplementary Figure S2A, only some of the DS isoforms decreased the viability of cancer cells moderately when they were applied at the highest concentration (25 µg/mL) compared to the control; however, this effect was strongly dependent not only on the glycan structure but also on the cell type. NF, which exerted a statistically significant impact on both luminal cancer lines (Figure 2A) was especially effective. In turn, PM markedly reduced the viability of only the BT-483 cells, whereas DF was able to decrease the viability of T-47D (Figure 2A) although this effect was statistically insignificant (*p* = 0.19). In contrast, no DS variant affected the viability of HCC-38 after either 24 h (Supplementary Figure S2A) or 48 h of treatment (data not shown). In order to investigate whether the observed effects on the cancer cell viability could lead to alterations in the number of cancer cells by modulating their proliferation, we examined the DNA biosynthesis in the cultures that were grown for 24 and 48 h exposed to the tested DS variants. The trends as to the cancer cell proliferation that was found in these cultures were similar regardless of the length of the exposure time. Thus, to illustrate the persistence of the induced effects, we decided to show the data that concern the longer incubation period. These results clearly demonstrate (Figure 2B and Supplementary Figure S2B) that some of the structural DS isoforms such as NF and PM at a concentration of 25 µg/mL were able to effectively decrease not only the viability of BT-483 cells but also their proliferation compared to the control. Moreover, NF when applied at this concentration also exhibited a strong inhibitory effect on the DNA synthesis in the T-47D cells, but this alteration was statistically insignificant compared to the control (Figure 2B). In turn, DF, which decreased (albeit statistically insignificantly) the viability of the T-47D cells, failed to affect their proliferation (Figure 2B). Furthermore, none of the tested variants affected the DNA biosynthesis in the HCC-38 cultures (Supplementary Figure S2B). To verify the data from the proliferation experiment in the luminal breast cancer cells, we examined the number of cells in the BT-483 and T-47D cultures that had been treated for 48 h with the DS variants exhibiting an inhibitory or non-inhibitory effect on the DNA biosynthesis. These variants were applied at a concentration of 25 µg/mL. Moreover, NF and PM were also used in the T-47D cultures at a concentration of 2.5 µg/mL, because the results of the proliferation test suggested that the variants can (insignificantly) stimulate the DNA biosynthesis under these conditions. The obtained data (Figure 2C) confirmed that NF and PM can significantly reduce the number of BT-483 cells compared to the control. In contrast, both isoforms only insignificantly decreased the cell count in the T-47D cultures (Figure 2C). However, unexpectedly, the most pronounced effect on these cells was exerted by DF, which reduced their number by almost two-fold compared to the control (Figure 2C). These results suggest that compared to the other DS variants, DF can induce processes that have the most persistent consequences for the cell number in T-47D cultures. However, these processes can probably also stimulate proliferation in some of the T47D cells, which could explain the observed lack of differences in the DNA biosynthesis between the control and DF-treated cultures (Figure 2B). On the other hand, the quantification of cellularity also showed that at a concentration of 2.5 µg/mL, neither NF nor PM had an effect on the number of T-47D cells (Figure 2C).

### 3.3. Short-Term Impact of the DS Variants on the Induction of Death in the Luminal Breast Cancer Cells

All of the above-mentioned data indicated that free DS chains with some structural properties can initiate processes in the luminal breast cancer cells during a 24-h exposure, the effects of which persist for at least 48 h and affect various aspects of cancer cell activity. Thus, in order to identify these quickly activated processes, we assessed the effect of the selected DS variants on the induction of cell death in the BT-483 and T-47D cultures that had been grown for up to 24 h exposed to these molecules. We decided to verify this possibility because the existence of a clear relationship between DS and apoptosis has recently been well documented in various cell lines [13,18]. Furthermore, at this stage of our experiment, we focused especially on those DS variants that had been exhibited a significant impact on the cancer cells in the viability and cell count tests, i.e., NF and PM for BT-483 and DF for T-47D. However, to the investigation in the T-47D cultures, we also included PM, which had no visible effect on either their viability or cell number. This step enabled us to gain insight into the differences between BT-483 and T-47D in their response to PM in those previous tests.

**Figure 3:**
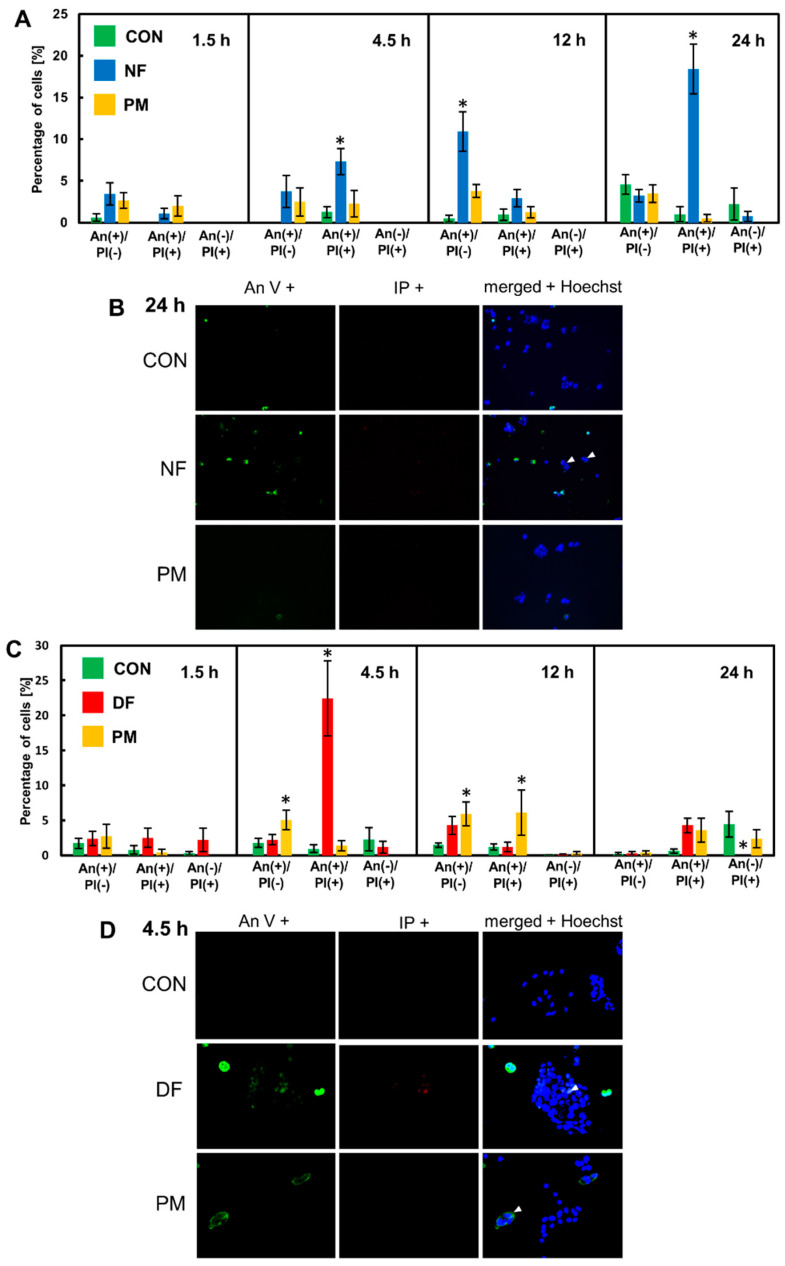
The DS variants accomplish their effect on the viability and count of the luminal breast cancer cells via the rapid induction of programmed cell death. (**A**,**C**) The BT-483 (**A**) and T-47D (**C**) cells were grown for the indicated time periods with NF or PM and with DF or PM, respectively, that had been used at a concentration of 25 µg/mL. The cells that were undergoing death were indicated by Annexin V binding (AnV(+) cells) and/or staining with propidium iodide (PI(+) cells). The nuclei of all cells were stained with Hoechst dye. Both living and dying cells were counted in at least ten non-overlapping fields from each of two independent experiments. The results are expressed as the percentage of dying cells and are presented as the mean ± SEM that was calculated for all of the obtained images. *—difference statistically significant (*p* ≤ 0.05) versus the control. (**B**,**D**) The representative images illustrating the most pronounced effects on AnV and/or PI binding in the BT-483 (**B**) or T-47D (**D**) cells that were exposed for the indicated time periods to NF or DF, respectively. Arrowheads indicate large nuclei in the dying cells. The images were taken at a magnification of ×400.

In the cultures that had been grown in the presence of the tested variants for several time periods, we found an increase in both the Annexin V (AnV) binding to the plasma membrane and in the nuclear uptake of propidium iodide (PI) compared to the control (Figure 3B and D and Supplementary Figure S3). Moreover, almost all of the dying cells in the treated cultures had an AnV+ phenotype, which suggests that they died via the programmed process. Notably, in the cultures that had been exposed to NF, and especially to DF, the dying cells had preserved or even enhanced the size of their nuclei and had formed distinct clusters or large foci (Figure 3B,D). Calculations of the percentages of the AnV-positive and/or PI-positive cells revealed that two DS variants, i.e., NF and DF strongly stimulated cell death in the BT-483 (Figure 3A) and T-47D (Figure 3C) cultures, respectively. However, these processes had different dynamics. NF progressively increased the number of AnV-positive cells over the incubation period starting at 4.5 h (Figure 3A). In turn, DF exerted a single-peak stimulatory effect that was observed after 4.5 h of treatment (Figure 3C). In contrast to NF and DF, PM manifested unexpected effects in both breast cancer cell lines. Despite the fact that this variant significantly reduced both the viability and cell number in the BT-483 cultures, it was unable to cause any significant effect on the dying cell number in these cultures during the entire incubation period (Figure 3A), although at 4.5 and 12 h of treatment, there was a statistically insignificant ~two-fold increase in the AnV-positive cell number compared to the control. On the other hand, PM had a neutral or poor impact on the cell viability and count in the T-47D cultures, but markedly increased the number of dying cells in these cultures compared to the control at both 4.5 h and 12 h of the incubation (Figure 3C). However, these alterations were a few-fold less than those that were induced by DF at 4.5 h of the treatment (Figure 3C). These results indicate that some DS variants can quickly trigger processes in the breast cancer cells that lead to the programmed cell death of these cells in a structure-specific and/or cell type-specific manner.

**Figure 4:**
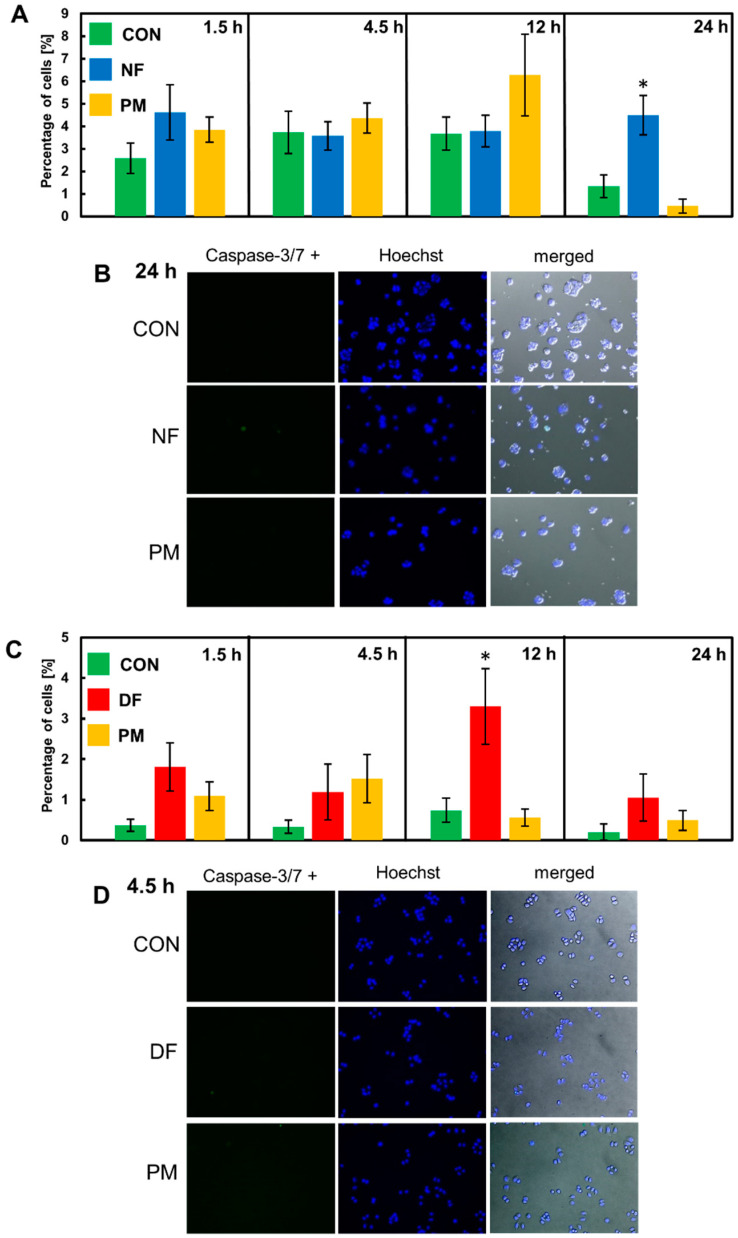
Apoptosis is not the predominant form of programmed cell death that is rapidly triggered by the tested DS variants in the luminal breast cancer cell cultures. (**A**,**C**) The quantification of caspase-3/7-positive cells in the BT-483 (**A**) and T-47D (**C**) cultures that were grown exposed to NF and PM or DF and PM, respectively, for the indicated time periods. The number of the cells exhibiting caspase-3/7 activity was estimated in at least ten non-overlapping fields from each of two independent experiments. The results are expressed as a percentage of the apoptotic cells and are presented as the mean ± SEM for all of the obtained images. *—difference statistically significant (*p* ≤ 0.05) versus the control. (**B**,**D**) The representative images that show the number of caspase-3/7-positive cells in the BT-483 (**B**) or T-47D (**D**) cultures grown in the exposure to NF or DF, respectively for the time periods in which the maximal AnV binding had been observed. Caspase-positive cells are indicated by green fluorescence. The images were taken at a magnification of ×200.

In order to investigate whether apoptosis was really this form of programmed cell death that was initiated by the tested variants of DS in the luminal breast cancer cells during the short-term incubation, we examined the activity of the so-called effector caspases (caspase-3/7) in the cultures that had been exposed to these molecules for the same time periods as in the cell death assay. A quantitative analysis of the obtained images (Figure 4B,D and Supplementary Figure S4) revealed that NF markedly increased the percentage of BT-483 cells that had caspase-3/7 activity compared to the control only at 24 h of the incubation (Figure 4A), whereas DF exerted this effect in the T-47D cultures at 12 h of the treatment (Figure 4C). However, it should be emphasized that the percentage of caspase-3/7-positive cells in the cultures that had been exposed to NF or DF was a few-fold less than the total percentage of the cells that exhibited AnV binding in these cultures (Figure 3B,D and Supplementary Figure S4). In turn, PM did not affect the number of caspase-3/7-positive cells in the T-47D cultures compared to the control over the entire incubation period (Figure 4C), although it insignificantly increased the quantity of these cells in the BT-483 line that had been grown in its presence for 12 h (Figure 4A). The data above clearly indicate that apoptosis is not the only or even the major type of programmed death that can be triggered by the DS variants in luminal breast cancer cells during short-term exposure.

**Figure 5:**
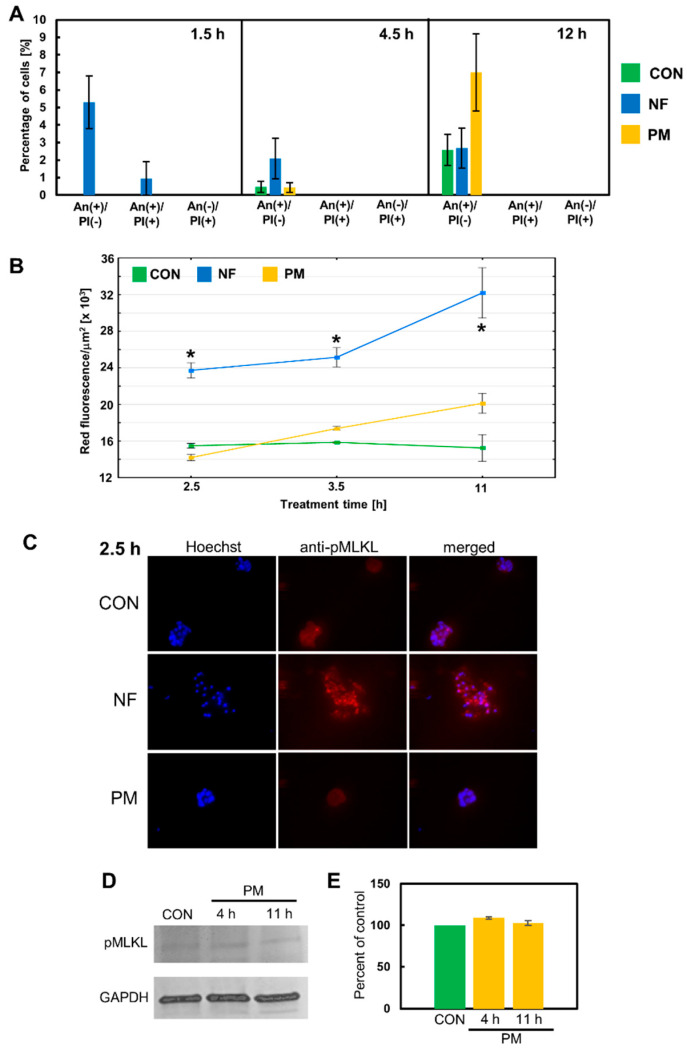
Necroptosis is a major form of programmed cell death that is rapidly induced by NF but not by PM in the BT-483 cancer cells. (**A**) Only NF-mediated death is sensitive to the necroptotic inhibitor necrosulfonamide (NSA). NSA at a concentration of 6 µM had been applied 30 min before adding NF or PM that were used at a concentration of 25 µg/mL and were incubated with the cells for the indicated time periods. Dying cells were indicated using Annexin V binding and/or staining with propidium iodide. The data are expressed as a percentage of the cells exhibiting Annexin V and/or propidium iodide staining and are presented as the mean ± SEM that was calculated for all of the images that were obtained in two independent experiments. *—difference statistically significant (*p* ≤ 0.05) versus the control. (**B**) The dynamics of MLKL activation/oligomerization in BT-483 cultures that were exposed to NF or PM for the indicated time periods. The MLKL activation was estimated using the anti-phospho-MLKL antibody. The measurement of fluorescence was conducted for the bodies of all of the cells that were present in at least six non-overlapping fields from each of two independent experiments. The results are expressed as the mean red fluorescence value per µm^2^ ± SEM. *—difference statistically significant (*p* ≤ 0.05) versus the control. (**C**) Representative images that show the most prominent effect of NF but not PM on the MLKL activation (magnification, ×400). (**D**) PM is unable to induce the activation of MLKL in the BT-483 cells. The representative western blot analysis of PM-mediated effect on the level of phospho-MLKL in BT-483 cells that were exposed to this variant for the indicated time periods. (**E**) Quantitative analysis of the obtained immunoblots illustrates the inability of PM to induce the MLKL activation in the BT-483 cells. The levels of phospho-MLKL were normalized to GAPDH content. The results are expressed as the mean ± SD of three independent experiments.

**Figure 6:**
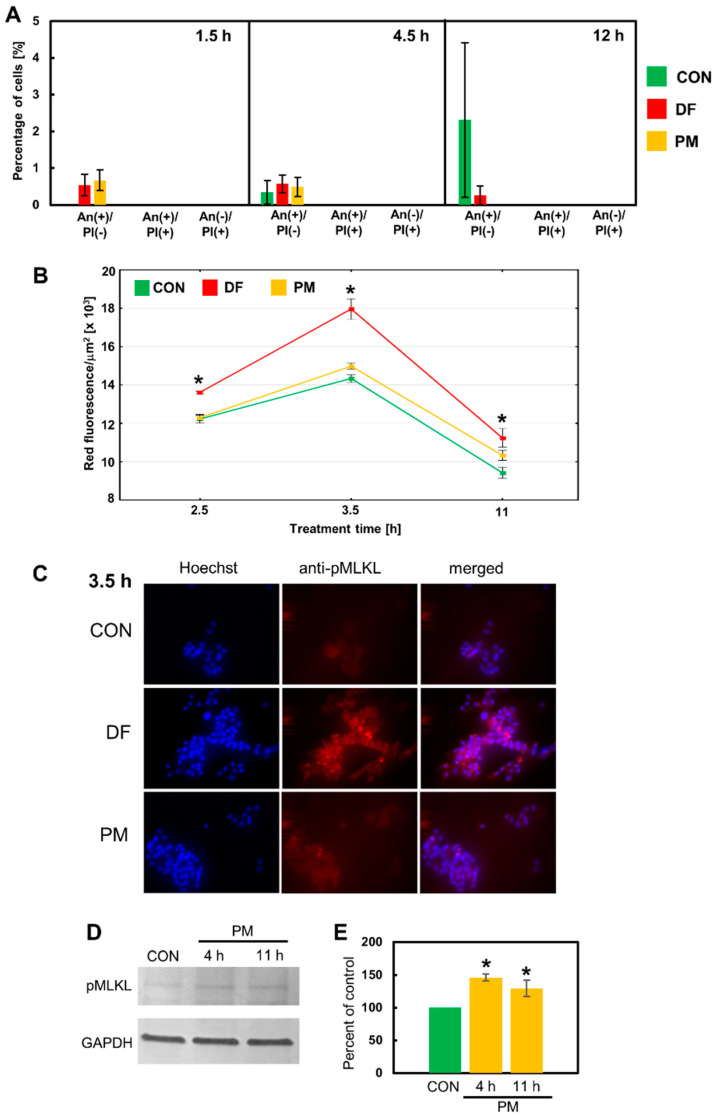
Necroptosis is at least a major type of programmed cell death that is quickly triggered by DF or PM in the T-47D cells as results from (**A**) a significant inhibitory effect of NSA on the number of dying cells in the exposed cultures and from the ability of these variants to induce the activation of MLKL, which was detected by immunofluorescence (**B**,**C**) or immunoblotting (**D**,**E**) using the anti-phospho-MLKL (S358) antibody. All of the above experiments were performed as described in the legend in Figure 5. (**A**) The data are expressed as a percentage of the cells exhibiting Annexin V and/or propidium iodide staining and are presented as the mean ± SEM that was calculated for all of the images that were obtained in two independent experiments. (**B**) The results are expressed as the mean red fluorescence value per µm^2^ ± SEM. *—difference statistically significant (*p* ≤ 0.05) versus the control. (**E**) The results are expressed as the mean ± SD of three independent experiments. *—difference statistically significant (*p* ≤ 0.05) versus the control.

The phenotypical features of the breast cancer cells that died in response to the DS variants such as AnV binding, the lack of caspase-3/7 activity, and the presence of large, often visibly swollen nuclei suggest that these cells might undergo a necroptotic death. In the classical pathway of this lytic form of programmed death, which is induced after the activation of death receptors such as receptor-1 for tumor necrosis factor alfa (TNF-alfa), two receptor-interacting serine/threonine protein kinases (RIPK)-1 and -3 assemble into a cytosolic oligomeric complex that is called a necrosome [19]. Within this complex, the activated RIPK-3 phosphorylates the effector component of necroptosis—mixed lineage kinase domain-like pseudokinase (MLKL) [19]. Then, the activated MLKL oligomerizes and is translocated to the plasma membrane, where it perforates this membrane [19]. Moreover, phosphorylated MLKL is also an executor of the necroptosis that is triggered via alternative signaling after the activation of the Toll-like receptors 3 and 4 [20]. However, the execution of necroptosis can be inhibited by compounds such as NSA, which is believed to block the MLKL transfer into the plasma membrane [21]. Therefore, we used NSA at a concentration that did not significantly reduce the cell number in the control cultures in our preliminary experiment, in order to determine whether this inhibitor might affect the DS variant-mediated induction of programmed death in the breast cancer cell lines. In this examination, we focused only on the short-term (up to 12 h) exposure of BT-483 and T-47D to the variants because the majority of them demonstrated their stimulatory effects on programmed cell death during such a period (Figure 3). The application of NSA eliminated any statistically significant increases in the percentage of AnV-positive cells that had been observed in BT-483 and T-47D cells exposed to NF or DF/ PM, respectively, for 4.5 and/or 12 h (Figure 5A and Figure 6A vs. Figure 3). Thus, the obtained data suggest that necroptosis might be the predominant, if not the only form of programmed death that is induced by at least some structural variants of DS during their short-term action on luminal breast cancer cells. In order to further corroborate this hypothesis, we examined the presence of activated MLKL oligomers in the breast cancer cells that had been exposed to the DS variants for three time periods. Such complexes were detected in single cells of the control cultures in which they formed either delicate linear structures extending along the cell membrane (BT-483 cells) (Figure 5C and Supplementary Figure S5A) or large light clusters that were located peripherally in the cell bodies (T-47D cells) (Figure 6C and Supplementary Figure S5B). It is intriguing whether such localization of phosphorylated MLKL might regulate the permeability of the cell membrane in the control cells thereby facilitating intercellular communication. On the other hand, the exposure of the cancer cells to NF or DF brought about a marked increase in the fluorescence signal (Figure 5C and Figure 6C and Supplementary Figure S5), which suggests the intensification of both the MLKL activation and oligomerization, although the dynamics (Figure 5B and Figure 6B) of this process and its nature (Figure 5C and Figure 6C) were strongly dependent on the variant structure and cell type. NF generated the appearance of many fine-grained fluorescence-active structures in BT-483 cells at 2.5-h incubation (Figure 5C). These structures were most likely able to form larger aggregates that were apparent in the coexistence with the former in the cell cultures after 3.5 h of treatment (Supplementary Figure S5A). In addition, the occurrence of both types of these anti-phospho-MLKL-reactive structures also persisted after 11 h of treatment (Figure 5B and Supplementary Figure S5A). Notably, although the NF-triggered activation/oligomerization of MLKL was visible in almost all of the BT-483 cells, especially after 2.5 and 3.5 h of treatment, these cells differed markedly in respect to the intensity of their responsiveness (Figure 5C and Supplementary Figure S5A). In turn, DF mainly induced the formation of larger-size fluorescence-active structures in T-47D cells (Figure 6C and Supplementary Figure S5B). More-over, this process was most pronounced after 3.5 h of incubation and often affected the neighboring cells (Figure 6B,C), which overlapped with the focal occurrence of AnV–positive cells in these cultures (Figure 3D). However, it should be emphasized that regardless of the DS variant that was used, the activation of MLKL that was induced by it was time-correlated with programmed cell death, and clearly preceded the manifestations of this death in both the BT-483 and T-47D cells (Figure 5B and Figure 6B vs. Figure 3).

In contrast, immunocytochemical analysis of the MLKL activation in the BT-483 and T-47D cells that were exposed to PM did not provide any clear answer as to the nature of the programmed cell death that was triggered by this variant (Figure 5B and Figure 6B) mainly due to the low level of the induced fluorescence signal. Nevertheless, based on the results of both the effector caspase activity test and the experiment with NSA it can be assumed that PM can induce apoptosis in the BT-483 cells and necroptosis in the T-47D cells during its short-term action. Therefore, in order to resolve this issue, we compared the levels of phospho-MLKL in these cell lines that had been treated with PM using immunoblotting analysis. The obtained data (Figure 5D,E and Figure 6D,E) indicate that both breast cancer lines had some basal level of MLKL activation, which was observed by the detection of a phospho-MLKL band in the extracts from the control cultures. On the other hand, the immunoblot analysis clearly showed that PM was able to increase the level of the activated MLKL only in the T-47D cells during both the 4 h and 11 h treatments (Figure 6D,E vs. Figure 5D,E). Thus, this observation supports the suggested differences in the response to PM between the T-47D and BT-483 cell lines.

### 3.4. Short-Term Effects of the DS Variants on the Mitochondrial Function and Oxidative Stress in the Cytoplasm of Breast Cancer Cells

**Figure 7:**
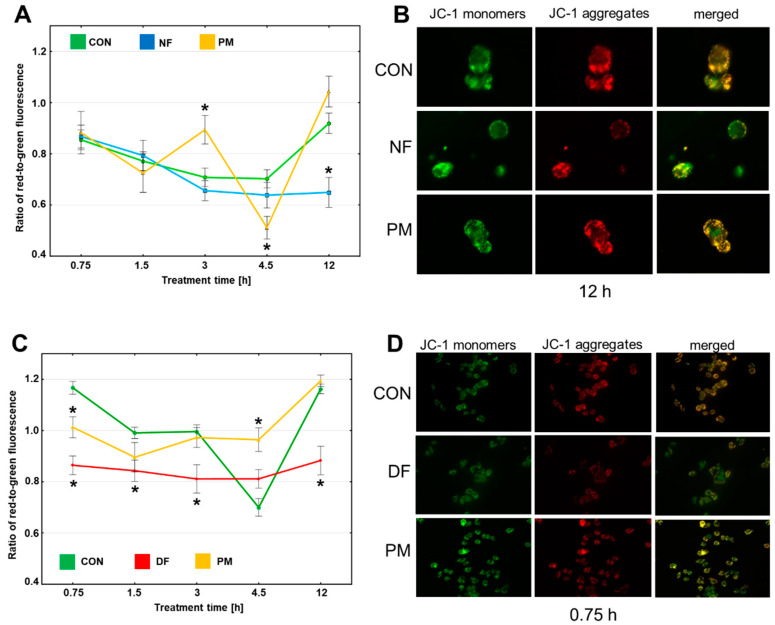
The variants of DS can induce rapid alterations in the mitochondrial transmembrane potential (∆Ψm) of the luminal breast cancer cells in a manner that is dependent on the structure of these molecules and cell type. (**A**,**C**) The dynamics of ∆Ψm in the BT-483 (**A**) and T-47D (**C**) cells that were cultured in the presence of NF and PM or DF and PM, respectively, for the indicated time periods. ∆Ψm was estimated as a ratio of red-to-green fluorescence, which is dependent on the mitochondrial membrane status-mediated polymerization of JC-1. The measurement of fluorescence was conducted for the bodies of all of the cells that were present in at least ten non-overlapping fields from each of two independent experiments. The results are expressed as the mean ±SEM. *—difference statistically significant (*p* ≤ 0.05) versus the control. (**B**,**D**) Representative images that show the most prominent effects of the tested DS variants on ∆Ψm in the cultures of BT-483 (**B**) and T-47D (**D**) cells. The images were taken at a magnification of ×400.

In order to investigate the mechanism(s) that are triggered by the DS variants in the breast cancer lines and are potentially up-stream of the induction of necroptosis, we assessed the function of the mitochondrion in the BT-483 and T-47D cells during their short-term (up to 12 h) exposure to the glycans by measuring the transmembrane potential ∆Ψm. A reduction in ∆Ψm, which is detected as a decrease in the ratio between the red and the green fluorescence, is a reflection of the phenomenon that is called the mitochondrial permeability transition. This process represents a key mechanism that is implicated in the induction of several forms of programmed death such as apoptosis or necroptosis [22]. A quantitative analysis (Figure 7A,C) of the obtained images (Figure 7B,D and Supplementary Figure S6) showed that the exposure of BT-483 cells to NF led to a progressive reduction in ∆Ψm that began at 3 h of treatment and was statistically significant compared to the control at 12 h of the incubation period (Figure 7A) These changes in the mitochondrial function occurred in parallel with an increase in the percentage of the AnV-positive and NSA-sensitive cells in these cultures (Figure 7A vs. Figure 3A and Figure 5A). In turn, the PM that did not significantly affect the percentage of the AnV-positive BT-483 cells (Figure 3A) was still able to trigger ∆Ψm pulsations (Figure 7A), which were manifested as alternations of the reductions (significant at 4.5 h of the incubation period compared to the control) and increases (significant at 3 h of the treatment compared to the control) of this parameter. The open question is whether this pulsatory manner of the PM-mediated changes in ∆Ψm might be associated with the lysosomal processing of this variant in the BT-483 cells and the liberation of unknown biologically active sequences from its chains. In turn, the presence of PM in the T-47D cultures after an initial, statistically significant reducing effect on ∆Ψm had a neutral or even stabilizing impact on this parameter over the remaining exposure period compared to the control (Figure 7C). The observed dynamics of the ∆Ψm alterations were poorly correlated with the mode of the occurrence of the AnV-positive and NSA-sensitive cells in the T-47D cultures that had been exposed to PM (Figure 7C vs. Figure 3C and Figure 6A). In contrast, the exposure of T-47D to DF caused a significant decrease in ∆Ψm of these cells compared to the control over almost the entire period of the treatment (Figure 7C). However, these alterations in the mitochondrial function were also poorly associated with the dynamics of the DF-mediated induction of necroptosis in the T-47D cells (Figure 7C vs. Figure 3C and Figure 6A). Thus, it can be concluded that DS clearly affects the mitochondrial function in the luminal breast cancer cells in both a structure-dependent and cell-type-dependent manner. However, there were no close mutual relationships between the DS variant-mediated short-term effects on ∆Ψm and on the induction of necroptosis, especially in the T-47D cultures.

**Figure 8:**
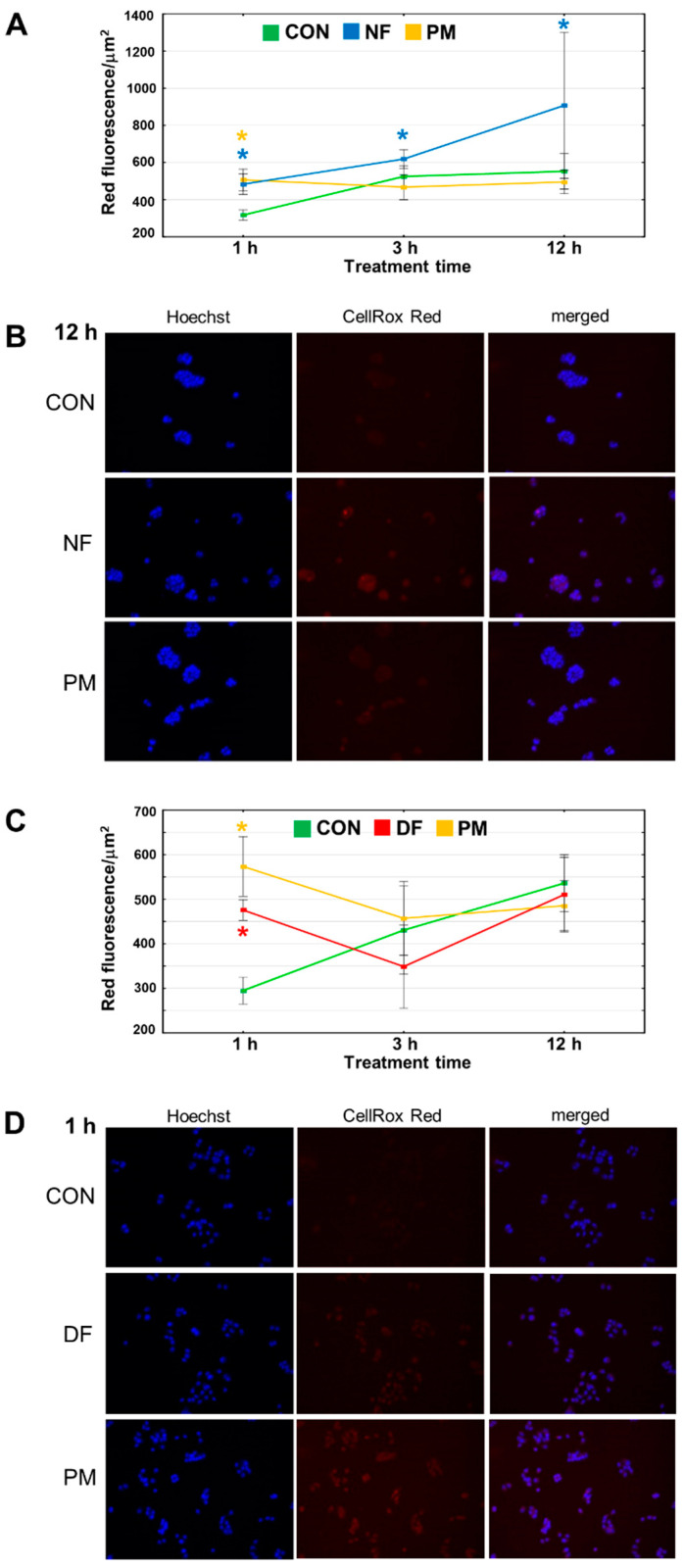
The DS variants can induce oxidative stress in the cytoplasm of the luminal breast cancer cells. (**A**,**C**) The dynamics of oxidative status in the BT-483 (**A**) and T-47D (**C**) cultures that grew in the exposure to NF and PM or DF and PM, respectively, for the indicated time periods. Red fluorescence was calculated from the bodies of all of the cells that were present in at least ten non-overlapping fields from each of two independent experiments. The results are expressed as the mean red fluorescence value per µm^2^ ± SEM. *—difference statistically significant (*p* ≤ 0.05) versus the control. (**B**,**D**) Representative images that show the most prominent effects of the tested DS variants on oxidative stress in the cytoplasm of BT-483 (**B**) and T-47D (**D**) cells. The images were taken at a magnification of ×200.

It has recently been found that the reactive oxygen species (ROS)-mediated oxidation of RIPK-1, which is a cytosolic molecule, can trigger necroptosis [23]. Thus, to further explore the mechanism(s) that can be up-stream of the induction of necroptosis and that might be rapidly triggered by DS in breast cancer cells, we examined the dynamics of oxidative stress in the cytoplasm of BT-483 and T-47D that had been exposed to the tested variants of this GAG for up to 12 h. The quantitative analysis (Figure 8A,C) of the obtained data (Figure 8B,D and Supplementary Figure S7) clearly indicated that all of the tested variants are able to rapid (visible after 1 h of treatment) induction of oxidative stress in the cytoplasm of both cancer lines. It is noteworthy that this phenomenon temporarily preceded the DS-triggered alterations in ∆Ψm in the BT-483 cells (Figure 8A vs. Figure 7A), but co-existed with the DS-dependent compromising of the mitochondrial function in the T-47D cells (Figure 8C vs. Figure 7C). This observation suggests that the DS-mediated overproduction of ROS in the cytoplasm might lead to mitochondrial discrimination in at least in BT-483 cells. However, after treatment of longer than 1 h, most of the tested DS variants lost their ability to generate oxidative stress in the cytoplasm of the BT-483 or T-47D cells (Figure 8A,C). Only NF supported or even increased the oxidative stress in the cytoplasm of the BT-483 cells throughout the entire exposure period (Figure 8A). A comparison between the dynamics of the DS variant-induced ROS overproduction in the cytoplasm and the dynamics of programmed death/necroptosis manifestations clearly indicated a similar manner of the occurrence of these phenomena albeit the first of them preceded the latter (Figure 8A vs. Figure 3A and Figure 5A as well as Figure 8C vs. Figure 3C and Figure 6A).

## 4. Discussion

Our results clearly demonstrate that the free chains of DF, NF, and/or PM at a high concentration are able to cause rapid and marked alterations in the activity of luminal breast cancer cells that lead to a moderate but statistically significant reduction in cell vi-ability and/or number, which persists for at least 24–48 h. The phenomenon that is directly responsible for these variant-mediated effects is the rapid induction of programmed cell death, probably starting before 4.5 h of treatment, which is the minimum time period for the onset of the first manifestations of this death. Such a conclusion can be drawn from the good quantitative correlation between the level of a decrease in the number of cancer cells and the percentage of AnV-positive cells in the cultures that had been exposed to most of these reactive DS variants. Interestingly, a close relationship between DS (and CS) and apoptosis, which is one of the forms of programmed cell death, was demonstrated in both normal and cancer cells [13,18,24,25]. This association can be briefly described as an anti-apoptotic action of CS and a pro-apoptotic effect of DS [13,18,24,25]. However, our study for the first time delivered direct evidence that some structural variants of DS such as DF, NF, and PM can also affect cancer cells by triggering their necroptosis. Nevertheless, it should be kept in mind that the variant-mediated induction of programmed cell death has a complex nature that depends on both the structural and cellular context. Some of the variants such as NF or DF were able to induce both necroptosis and apoptosis in the BT-483 or T-47D cells, respectively. This conclusion results from several observations. Firstly, the number of dying cells in the exposed cultures was highly sensitive to the action of the necroptotic inhibitor NSA, and both DS variants substantially stimulated the activation/oligomerization of MLKL, which is an executor of necroptosis. Moreover, both isoforms were also able to significantly increase the percentage of caspase-3/7-positive cells at certain time points of the treatment period. In turn, PM can most probably induce only one form of programmed death during short-term treatment. However, the nature of this cell response might be highly dependent on the breast cancer cell type. This suggestion is supported by our observation that when PM was applied to the T-47D cultures, it modestly but significantly stimulated the activation of the key necroptotic effector MLKL, but failed to affect the number of caspase-3/7-positive cells. In contrast, PM did not trigger a necroptotic stimulus in the BT-483 cells, as it results from a lack of a visible impact of this variant on the activation of MLKL in these cells. Unfortunately, we were unable to demonstrate that this isoform, despite its marked impact on the viability and cell count in the BT-483 cultures, can accomplish these effects via the induction of apoptosis. This might simply result from differences between the dynamics of PM-induced apoptosis in the BT-483 cells and the time protocol that we used to follow the phenomenon. There are some circumstances that suggest such an explanation. We observed that a ~few-fold (although statistically insignificant compared to the control) increase in the number of AnV-positive cells in the BT-483 cultures at 12 h of treatment was resistant to the action of the necroptotic inhibitor NSA. This increase was also time-correlated with a ~few-fold (although statistically insignificant compared to the control) increase in the number of caspase-3/7-positive cells in the exposed BT-483 cells. Thus, it is possible that PM might induce apoptosis in these cells during the interval between two checkpoints, i.e., 4.5 and 12 h of treatment. This suggestion is further supported by the finding that at 4.5 h of incubation, PM markedly decreased ∆Ψm in the BT-483 cells, which is a strong pro-apoptotic stimulus [22]. However, we cannot completely exclude that a PM-mediated reduction in the viability and cell count in the BT-483 cultures was triggered via mechanism(s) other than the induction of programmed death. On the other hand, our study also indicated that some structural variants of DS such as PS when used against BT-483 and T-47D or all of the tested isoforms when applied against HCC-38 failed to exert any apparent effect on the viability and proliferation (or number) of these cells. However, taking into account the fact that PM rapidly triggered necroptosis in the T-47D cells despite having no effect on the viability and number of these cells in the long term, caution should be exercised in concluding that the lack of detectable alterations in cell viability or number is equivalent to the complete lack of response of cancer cells to DS.

Our results demonstrated that even a very short (up to 1 h) exposure of luminal breast cancer cells to such DS variants as DF, PM, or NF can trigger several processes in these cells, the dynamics of which depends on the structure of the variants and cell type. These processes that precede the symptoms of programmed death in the exposed cells affect the mitochondrial function and oxidation-reduction balance in the cytoplasm, both of which are key upstream pro-apoptotic factors [22]. Furthermore, oxidative stress in the mitochondrial compartment was found to be an important trigger of necroptosis [23]. However, our results clearly show that the pattern of occurrence of necroptotic manifestation in the luminal breast cancer cells that were exposed to DF, PM, or NF is better correlated with the dynamics of ROS overproduction in the cytoplasm of these cells than with the dynamics of alterations in their mitochondrial transmembrane potential. This relationship allows for the assumption that an oxidation-reduction imbalance in the cytoplasm is rather an upstream mechanism on the induction of necroptosis in luminal breast cancer cells.

Our data support the previous reports [10,11,12,13] that some structural variants of (CS)/DS have an anti-cancer activity when they are used at high concentrations. Moreover, both our study and the majority of the above-mentioned studies [10,11,12,13] strongly indicate the importance of the cellular context in which these variants function. This phenomenon manifests as the promotion of anti-cancer activity only toward specific types of cancer cells [10,11,12,13]. For example, we observed that NF significantly and persistently affected the viability, proliferation, and cell number only in the BT-483 cultures, while it only exhibited transitory effects toward the T-47D cells. In turn, DF had the opposite tendencies. However, both variants had no visible effects on the HCC-38 cells. Thus, our observations confirm the known facts about the extreme heterogeneity of tumor cells in terms of their metabolism as well as their sensitivity to treatment. Nevertheless, our study clearly showed that the strongest anti-cancer stimuli in luminal breast cancer cells were generated by NF and DF, which share structural features with those that are characteristic of the DS remo-deling in the tumor microenvironment. Based on these observations, the remodeling of DS in the tumor niche might represent a defense mechanism of the host that is responsible for slowing down cancer growth. However, there are reports that show that alterations in the composition of DS from the tumor niche enable tumor growth, migration, and invasion [6,7]. On the other hand, we found that DS with a “normal” structure might also induce pro-survival signals in metastatic cancer cells as it had resulted from the preservation of the cell number in the T-47D cultures that had been exposed to PM for 48 h despite the rapid induction of moderately intensive necroptosis by this variant. The discrepancies in the observed response of the cancer cells to the DS variants, manifesting as the pro-cancer or anti-cancer effects of these molecules, might result from several reasons. It is possible that in a manner that is dependent on their concentrations and sequences, DS or the products of its extracellular processing might affect interactome at the cancer cell surface or—after endocytosis—interfere with various intracellular events as observed in mucopolisaccharidoses [18]. The substantial role in the cancer cell response can also play the molecular background in which they are grown because other ECM components generate strong anti- or pro-survival signals via integrin receptors [26], which might strongly modulate DS-induced effects. Finally, our study indicates that differences in DS variant-mediated biological effects might partly result from the ability of these molecules to induce necroptosis of different intensities. This death is believed to play a two-faced role in cancer growth. It has been shown that a well-functioning necroptotic pathway is necessary for the sensitivity of cancer cells to chemotherapeutics [27]. In contrast, a genetic knockout of the major necroptotic players such as RIPK-1, RIPK-3, or MLKL leads to a significant attenuation of tumor growth both in vitro and in vivo as well as to an increase in the radiosensitivity of cancer cells via a marked decrease in both the NF-κB activity and the secretion of pro-inflammatory cytokines [28]. Moreover, the lytic nature of necroptosis is associated with the induction of a local inflammatory response, which additionally creates survival stimuli for cancer cells. Interestingly, we found detectable levels of activated MLKL in the control cultures of both the BT-483 and T-47D cells, which might indicate the importance of the necroptotic pathway in sustaining the growth of these cells. Thus, necroptosis of low intensity such as that observed in the control cultures or in the T-47D cells that had been exposed to PM might rather represent a pro-survival stimulus for cancer cells. In contrast, a strong induction of this death seems to be responsible for a significant and persistent reduction in the cancer cell number although it can also trigger an increase in DNA biosynthesis in part of the cancer cell population, which was observed in the T-47D cell in the presence of DF. Moreover, it is possible that necroptosis of high intensity may be a factor that triggers apoptosis in breast cancer cells thereby creating an additional mechanism by which the reduction in the viability and number of cancer cells can be further sustained. This suggestion is supported by our observation that when inducing both forms of programmed cell death as was found during the short-term exposure of BT-483 or T-47D to NF or DF, respectively, the intense necroptosis preceded the occurrence of apoptosis. Furthermore, the relationship between necroptosis and apoptosis was also indicated by the observation that in the breast cancer cells that had been exposed to DF, the inhibition of the former death by NSA completely eliminated the increase in the percentage of AnV-positive cells thereby also suggesting the inhibition of this variant-mediated apoptosis.

Taken together, our study clearly shows that the free DS chains can markedly interfere with the biology of cancer cells even during short-term exposure. However, we merely sketch out the mechanisms that are associated with the DS variant-mediated induction of necroptosis. Thus, further investigations should be conducted in order to identify the receptor(s) and downstream signaling that is involved in the DS action. Moreover, it is extremely interesting how cancer cell function is affected by the DS variants that had no visible impact on the cancer cell viability, proliferation, or number. A similar question concerns the reactive DS variants when they are used at low concentrations. We also think that in order to have a good understanding of the DS-mediated impact on cancer cells, an estimation of the signaling that is induced in basic conditions such as cell cultures on plastic, should simply precede but not replace the use of more complex experimental models such as on matrigels or 3-D cultures. On the other hand, a distinct and serious problem is the lack of simple and quick methods for examining the DS sequence. Thus, using the DS variants that had structural features similar to those of the DS from the tumor niche, which was based on the profile and content of their disaccharides, we did not know whether all of these molecules also shared their sequences and the resulting binding properties.

## Data Availability

Not applicable.

## Supplementary Materials

The following supporting information can be downloaded at: https://www.mdpi.com/article/10.3390/cells11010173/s1, Figure S1: Typical chromatographic separation of the unsaturated disaccharides that were released after the degradation of the tested DS variants with chondroitinase ABC; Figure S2: DS variants-mediated effects on the viability (A) and DNA biosynthesis (B) in triple negative breast cancer cell line HCC-38; Figure S3: The DS variants significantly affect the number of Annexin V (AnV)-positive cells in the BT-483 (A) and T-47D (B) cultures at several time periods of the treatment; Figure S4: The number of caspase-3/7-positive cells was also very low in the cultures of BT-483 (A) and T-47D (B) cells that grown exposed to the tested variants for the remaining time periods in which AnV binding had been statistically increased compared to the control; Figure S5: The representative images that illustrate the DS variants-mediated impact on the activation of MLKL in BT-483 (A) and T-47D (B) cells that were exposed to these molecules for the remaining periods of treatment; Figure S6: The representative images that illustrate the DS variants-mediated impact on ΔΨm in BT-483 (A) and T-47D (B) cells that were exposed to these molecules for the remaining periods of treatment; Figure S7: The representative images that illustrate the DS variants-mediated effects on oxidative stress in the cytoplasm of BT-483 (A) and T-47D (B) cells that were exposed to these molecules for the other periods of treatment.
