# Supplementary material for: Dermatan Sulfate Affects Breast Cancer Cell Function via the Induction of Necroptosis
Source: Cells. 2022 Jan 5;11(1):173. doi: 10.3390/cells11010173 (PMC8750542; doi:10.3390/cells11010173)
Supplement: Supplementary file 1 [file cells-11-00173-s001.zip › cells-1501565-supplementary.pdf]

Supplementary material

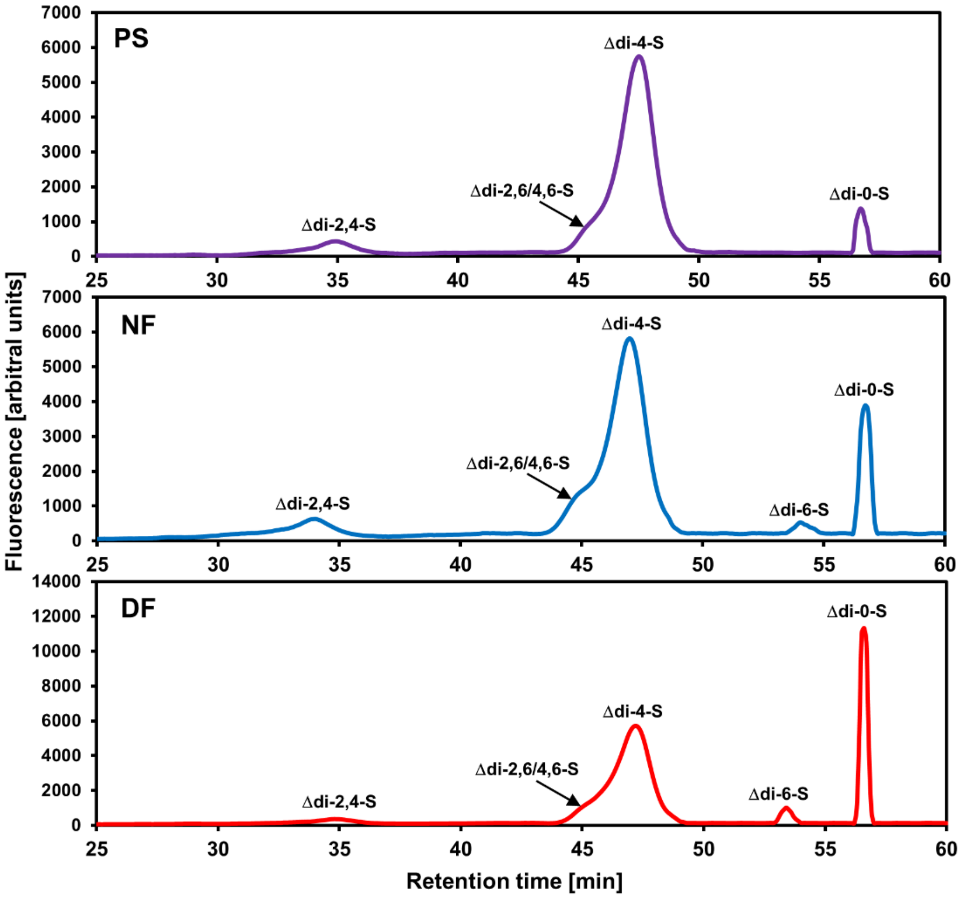

Figure S1. Typical chromatographic separation of the unsaturated disaccharides that were released after the degradation of the tested DS variants with chondroitinase ABC. PS – DS from porcine skin, NF – DS from normal human fascia, DF – DS from fibrosis affected fascia, PM – DS from porcine intestinal mucosa. Δdi-2,4-S – ΔHexA(2S)-GalNAc(4S); Δdi-2,6-S – ΔHexA(2S)-GalNAc(6S); Δdi-4,6-S – ΔHexA-GalNAc(4,6S); Δdi-4-S – ΔHexA-GalNAc(4S); Δdi-6-S – ΔHexA-GalNAc(6S); Δdi-0-S – ΔHexA-GalNAc(0S) (where ΔHexA – 4-deoxy-α-L-threo-hex-4-enepyranosyluronic acid; GalNAc – N-acetyl galactosamine)

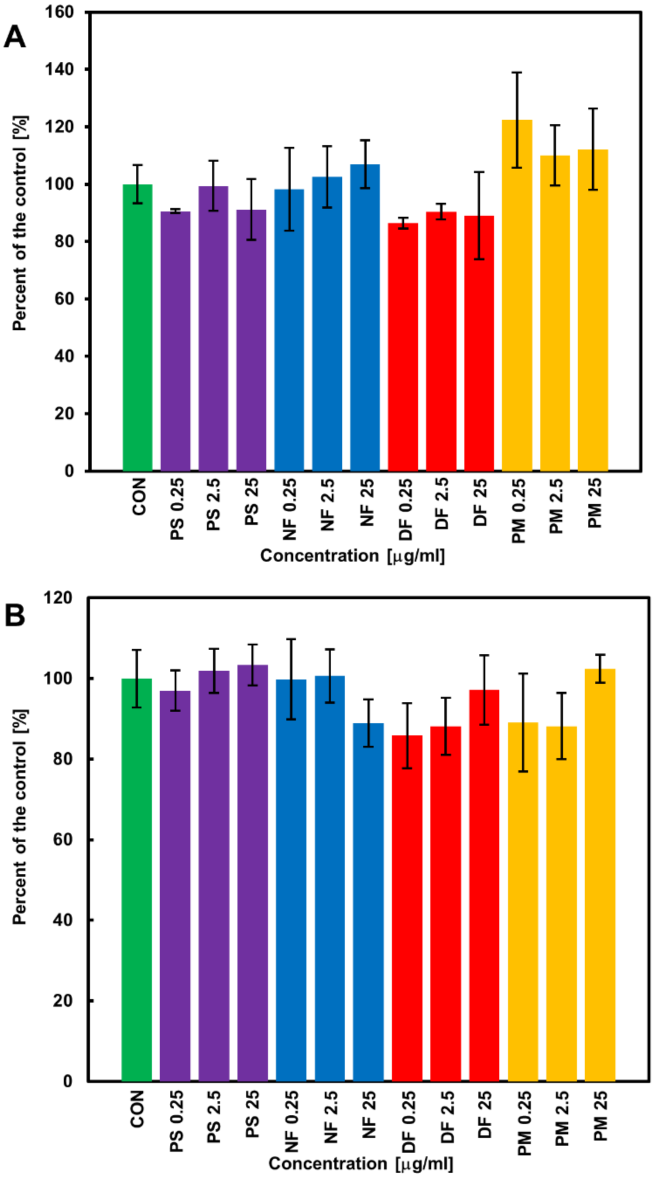

Figure S2. DS variants-mediated effects on the viability **(A)** and DNA biosynthesis **(B)** in triple negative breast cancer cell line HCC-38. **(A)** The cancer cells were exposed for 24 h to the structural variants of DS that had been applied at the indicated concentrations. Then, the cell viability was evaluated using the WST-1 test. **(B)** The cancer cells were grown for 48 h in the presence of the tested variants of DS that had been used at the indicated concentrations. The cell proliferation was evaluated by measuring the incorporation of the bromodeoxyuridine to DNA. The results are expressed as the percentage of effect that was visible in the control cultures and are presented as the mean ± SD of at least three independent experiments in which n = 3 for each DS concentration. CON – control (cultures untreated), PS – DS from porcine skin, NF – DS from normal human fascia, DF – DS from fibrosis affected fascia, PM – DS from porcine interstitial mucosa.

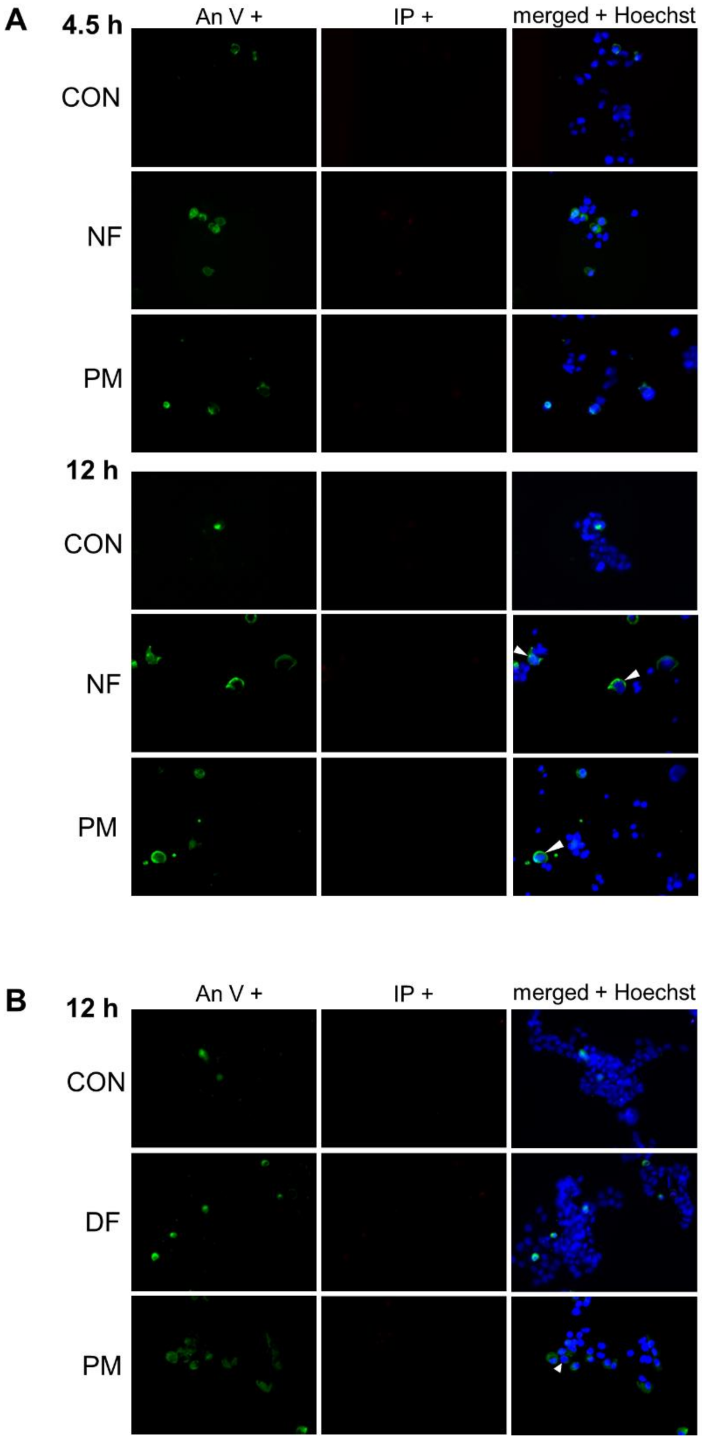

Figure S3. The DS variants significantly affect the number of Annexin V (AnV)-positive cells in the BT-483 (**A**) and T-47D (**B**) cultures at several time periods of the treatment. The representative images that show the AnV and/or PI binding in the cultures exposed to the tested variants for the indicated time periods. Arrow heads indicate large nuclei in the dying cells. The images were taken at a magnification of ×400.

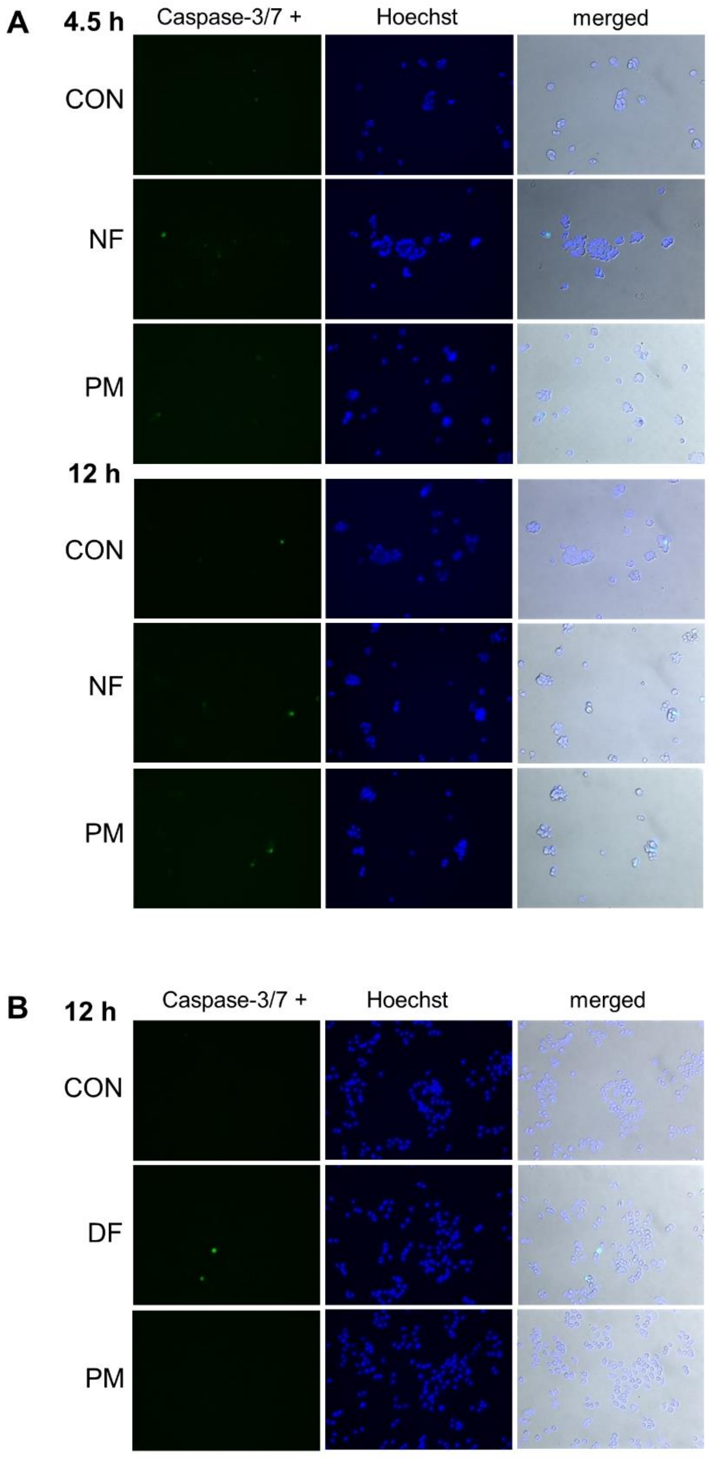

Figure S4. The number of caspase-3/7-positive cells was also very low in the cultures of BT-483 (**A**) and T-47D (**B**) cells that grown exposed to the tested variants for the remaining time periods in which AnV binding had been statistically increased compared to the control. The cells showing caspase activity are indicated by a green fluorescence. The images were taken at a magnification of ×200.

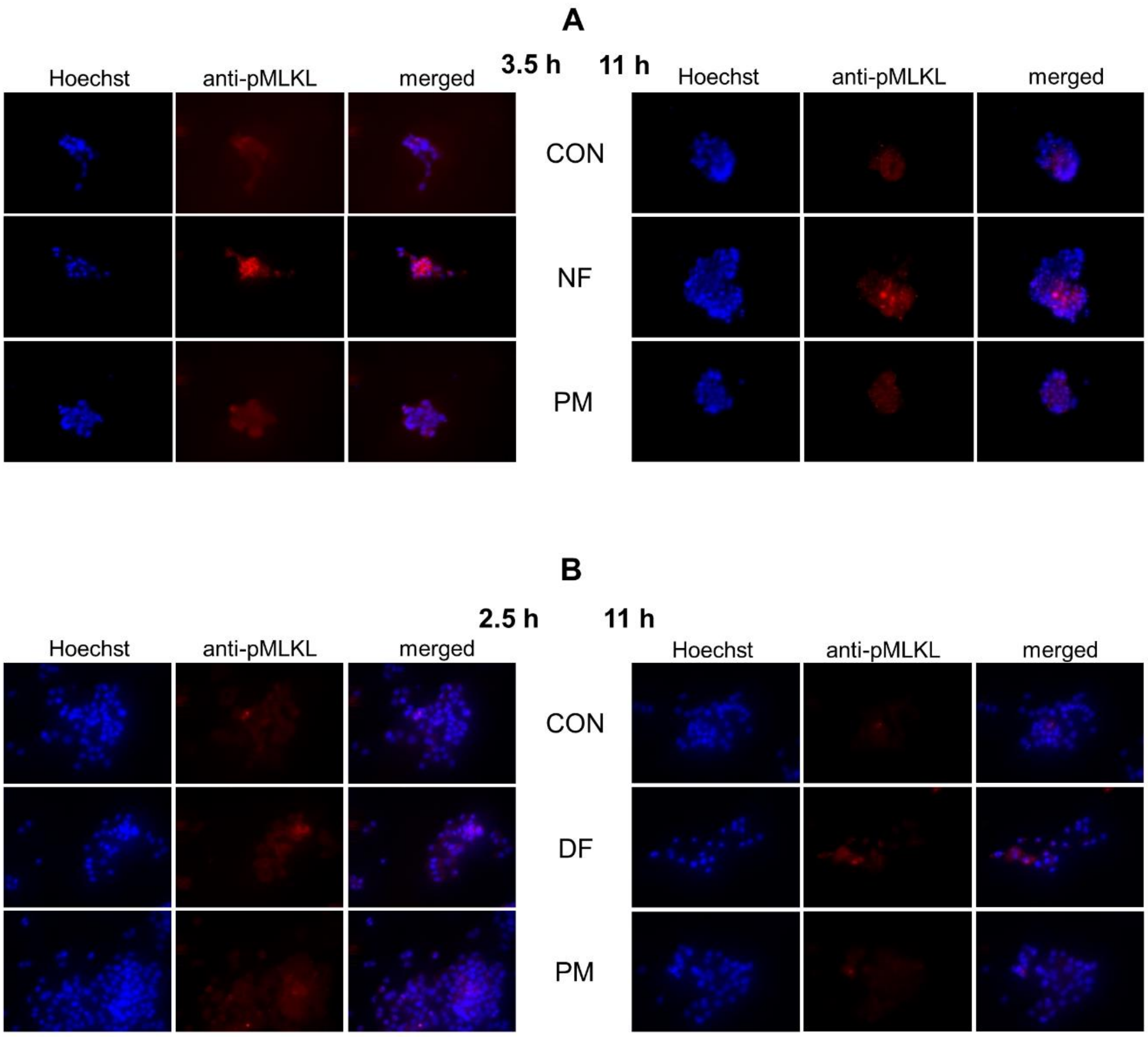

Figure S5. The representative images that illustrate the DS variants-mediated impact on the activation of MLKL in BT-483 (**A**) and T-47D (**B**) cells that were exposed to these molecules for the remaining periods of treatment.

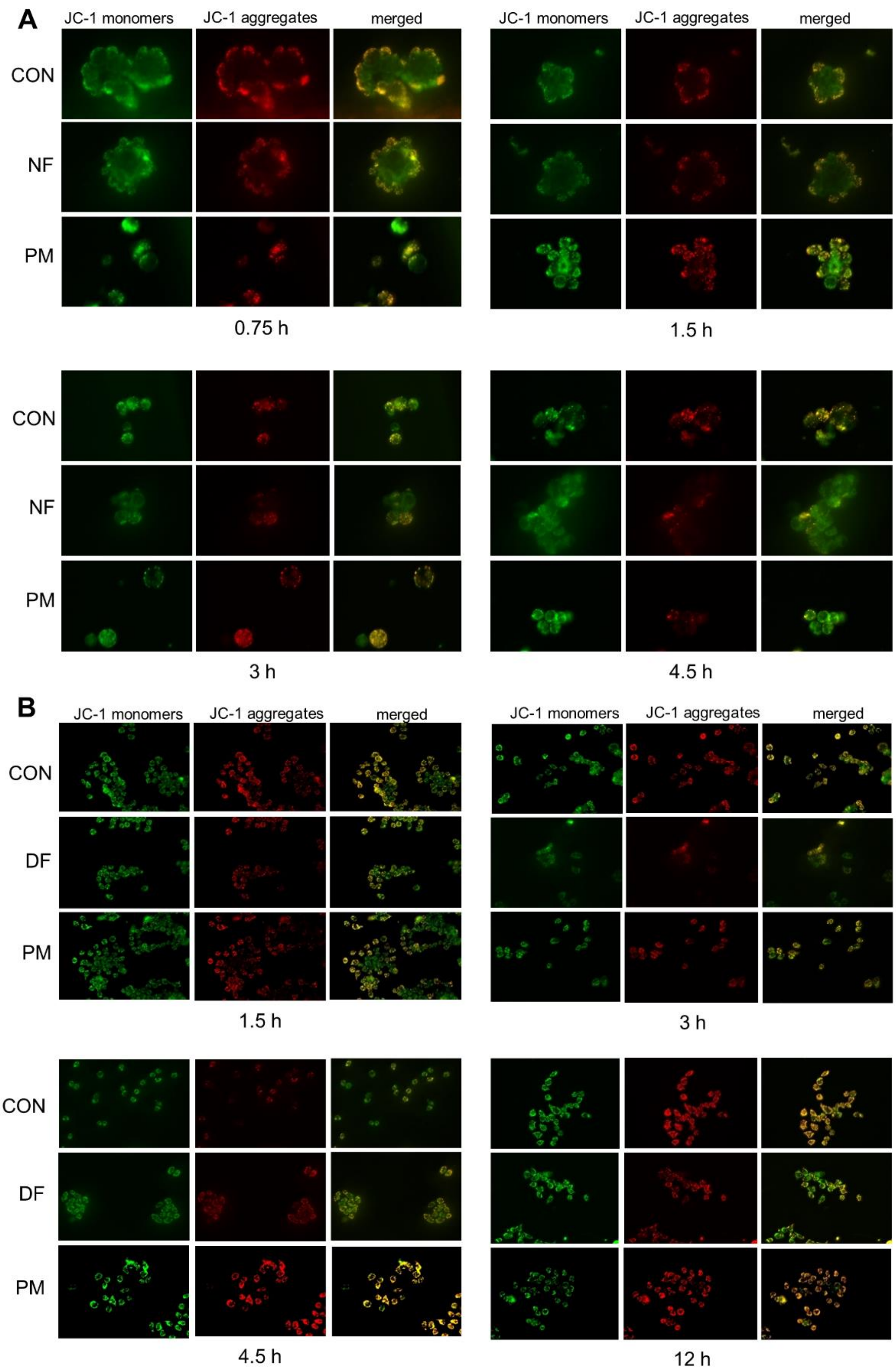

Figure S6. The representative images that illustrate the DS variants-mediated impact on $\Delta\Psi_m$ in BT-483 (**A**) and T-47D (**B**) cells that were exposed to these molecules for the remaining periods of treatment.

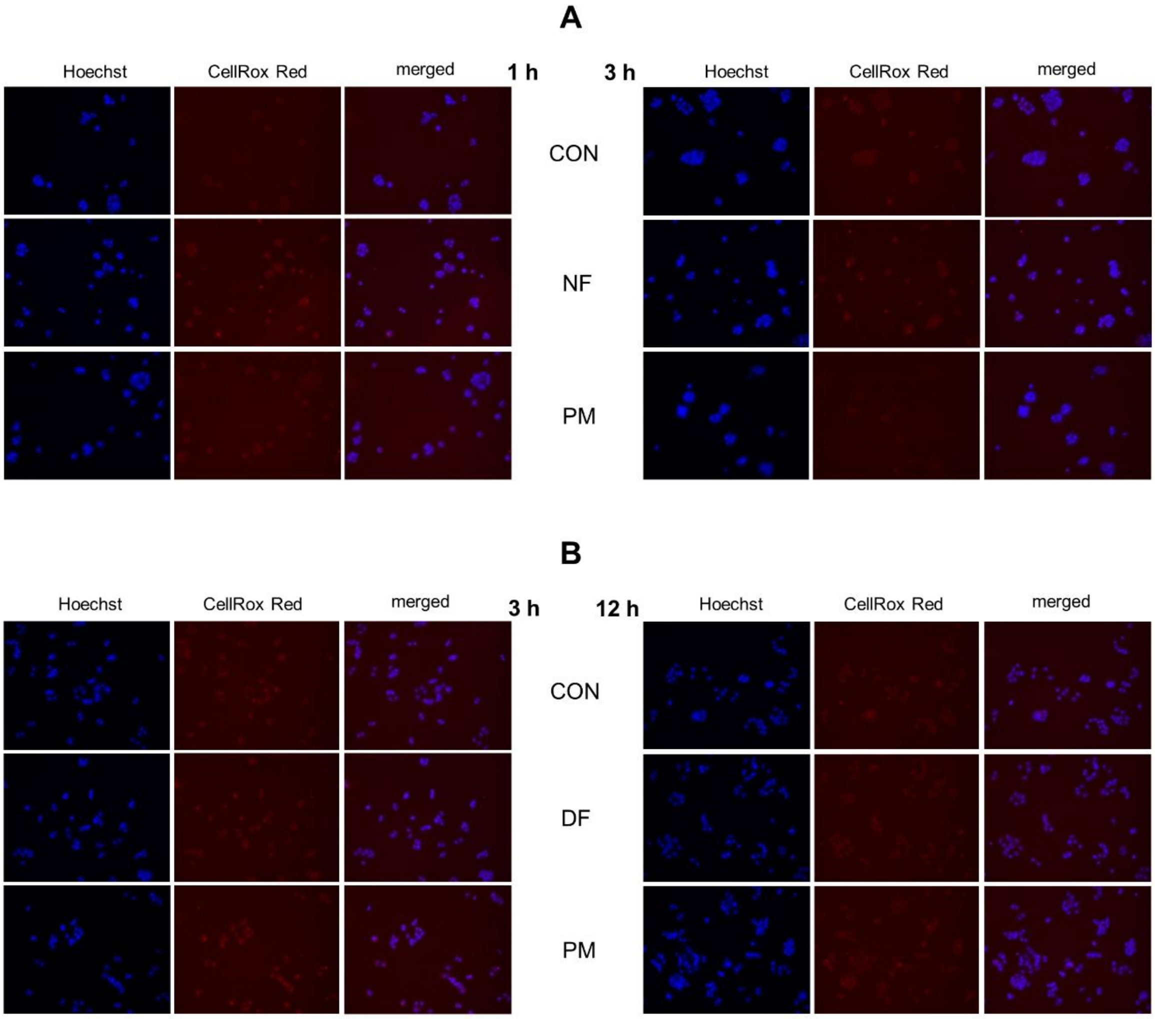

Figure S7. The representative images that illustrate the DS variants-mediated effects on oxidative stress in the cytoplasm of BT-483 (**A**) and T-47D (**B**) cells that were exposed to these molecules for the other periods of treatment.
